# Perceived social support and psychological wellbeing among vocational college students: the serial mediating roles of school belonging, academic self-efficacy, and psychological resilience

**DOI:** 10.3389/fpsyg.2026.1894510

**Published:** 2026-08-27

**Authors:** Qiyan Feng, Hanfeng Chen

**Affiliations:** 1 College of Digital Media, Chongqing Technology and Business Institute, Chongqing, China; 2 School of Creative Studies, Yiwu Industrial and Commercial College, Jinhua, China; 3 School of Computer Science and Technology, East China Normal University, Shanghai, China

**Keywords:** academic self-efficacy, perceived social support, psychological resilience, psychological wellbeing, school belonging, vocational college students

## Abstract

**Introduction:**

Psychological wellbeing is an important concern for vocational college students, who face academic demands, skills training, practicum requirements, and school-to-work transition pressures. This study examined whether perceived social support is associated with psychological wellbeing through a serial mediation process involving school belonging, academic self-efficacy, and psychological resilience.

**Methods:**

A cross-sectional online survey was conducted among vocational college students from Zhejiang, Chongqing, and Shanghai, China. A total of 500 questionnaires were collected, and 436 valid responses were retained for analysis. Participants completed self-report measures of perceived social support, school belonging, academic self-efficacy, psychological resilience, and psychological wellbeing. Data were analyzed using SPSS 27.0.1, AMOS 24, and supplementary Python scripts. Confirmatory factor analysis, structural equation modeling, and bootstrap mediation analysis with 5,000 resamples were conducted.

**Results:**

The five-factor measurement model showed good fit and outperformed alternative measurement models. The structural model also showed good fit to the data. Perceived social support was positively associated with school belonging, which was positively associated with academic self-efficacy, psychological resilience, and psychological wellbeing in sequence. Bootstrap results showed that the serial indirect effect through school belonging, academic self-efficacy, and psychological resilience was significant. The direct effect of perceived social support on psychological wellbeing also remained significant, indicating partial serial mediation.

**Discussion:**

These findings suggest that perceived social support is associated with vocational college students' psychological wellbeing both directly and indirectly through a theoretically grounded resource-transformation chain, in which external relational support is linked to school-contextual belonging, academic motivational belief, and adaptive psychological resilience. The study highlights the importance of strengthening students' social support, school belonging, academic confidence, and resilience in vocational education contexts.

## 1 Introduction

Psychological wellbeing has become an important concern in higher education (Harding et al., 2019), as students are increasingly required to cope with academic demands, interpersonal adjustment, career uncertainty, and rapid social changes. Rather than referring only to the absence of psychological distress, psychological wellbeing reflects positive psychological functioning, adaptive emotional experience, and the capacity to maintain a meaningful and satisfying life (Ryff, 1989, 2013). For vocational college students, this issue may be particularly important. Their educational experiences are closely connected with applied learning, skills training, practicum requirements, professional certification, and career preparation. These characteristics mean that their psychological wellbeing is shaped not only by general student life, but also by their ability to adapt to school-based learning, practice-oriented training, and the transition from school to work.

Perceived social support is widely recognized as an important protective resource for students' psychological adjustment and wellbeing. It refers to students' subjective perception that care, help, and encouragement are available from significant interpersonal sources, such as family members, friends, teachers, peers, or other important others (Zimet et al., 1988; McLean et al., 2023). For vocational college students, perceived social support may provide emotional reassurance, informational guidance, and practical assistance when they face learning pressure, skills-training challenges, and uncertainty about future employment. However, treating perceived social support only as a direct protective factor provides an incomplete theoretical explanation of its association with psychological wellbeing. The perceived availability of support does not by itself explain whether that support becomes psychologically embedded in students' educational experiences, whether it is connected with their confidence in managing academic and skills-related demands, or whether it is associated with their capacity to recover from setbacks. Students who perceive similar levels of support may therefore differ in psychological wellbeing depending on how that relational resource is connected with school-contextual, motivational, and adaptive psychological resources. A theoretically ordered process perspective is consequently needed to explain the functional pathway through which a relatively distal relational resource may be associated with psychological wellbeing.

This process-oriented question is particularly relevant in vocational education because its distinctive learning structure may make each stage of the proposed resource pathway especially salient. Vocational learning often depends on sustained teacher guidance, peer collaboration, and participation in classroom, workshop, and practicum communities. Under these conditions, perceived support may shape whether students experience themselves as accepted and legitimate members of the school community, making the connection between perceived social support and school belonging particularly relevant. Vocational education is also highly performance-oriented and feedback-intensive, involving repeated skills practice, practical assessment, and demonstration of occupational competence. Students who feel connected to the school community may be more willing to participate, seek feedback, collaborate with peers, and persist in these activities, thereby linking school belonging with academic self-efficacy. In addition, practical training, practicum preparation, and the school-to-work transition expose students to recurring setbacks and uncertainty. Confidence in managing concrete academic and skills-related demands may therefore be especially relevant to adaptive appraisal, strategic adjustment, and re-engagement, which are closely associated with psychological resilience. Because these pressures directly affect students' daily functioning and future occupational expectations, resilience may also be particularly relevant to psychological wellbeing. Thus, vocational education does not merely provide the setting for the proposed model; its relationally embedded, performance-oriented, and transition-focused characteristics help explain why the successive resource linkages may be especially salient (Hong et al., 2021; Liu et al., 2022; Liu, 2026).

Existing research has provided substantial evidence that perceived social support, school belonging, academic self-efficacy, and psychological resilience are each associated with student adjustment and wellbeing-related outcomes (Chu et al., 2010; Korpershoek et al., 2020; Zhang et al., 2024). Existing resource-based perspectives provide a general explanation of why valued resources may facilitate further resource gains. However, they provide limited specification of the psychological mechanisms through which resources located in different domains become connected within a particular educational context. In parallel, prior research on social support, belonging, self-efficacy, and resilience has often developed through relatively separate lines of inquiry or focused on individual and pairwise mechanisms. Such approaches clarify whether each resource is beneficial, but provide limited explanation of how relational availability, school-contextual connection, task-specific capability beliefs, and adaptive recovery may be functionally linked within a coherent process. The theoretical gap therefore concerns the mechanisms and functional differentiation through which resources across relational, contextual, motivational, and adaptive domains may become connected in explaining psychological wellbeing, rather than merely the absence of a study that includes all of these variables in one model.

To address this theoretical gap, the present study proposes a serial resource pathway linking perceived social support with school belonging, academic self-efficacy, and psychological resilience in a theoretically ordered sequence. Consistent with the resource-gain logic of Conservation of Resources theory, this ordering reflects differences in the functional roles of the resources (Hobfoll et al., 2018). Perceived social support is conceptualized as an external relational resource, whereas school belonging captures how students experience their connection with the educational environment. Academic self-efficacy represents a task-specific motivational resource concerning students' perceived capability to manage academic and skills-related demands. Psychological resilience is positioned as a more proximal adaptive resource because it concerns students' capacity to maintain or restore functioning when difficulties occur. The proposed sequence therefore moves from external relational availability to contextual connection, task-related confidence, and adaptive recovery. It is theoretically prioritized over other possible sequences because each resource is positioned according to its functional role and relative proximity to students' responses to academic and developmental pressure, although the proposed pathway is not assumed to be the only plausible mechanism.

Accordingly, the theoretical need for the present study lies in explaining how a relatively distal relational resource becomes connected with progressively more context-specific and proximal psychological resources, rather than merely confirming that perceived social support is associated with psychological wellbeing. On this basis, the proposed model offers three forms of conceptual significance. First, it reconceptualizes perceived social support from a stand-alone protective factor as a relational resource whose psychological significance may depend partly on whether it becomes embedded in students' experience of the educational environment. This perspective highlights that the perceived availability of support and the experience of being accepted within the school community are related but functionally distinct aspects of students' resource environment. Second, the model differentiates school belonging, academic self-efficacy, and psychological resilience according to the distinct functions they perform in students' adaptation: contextual membership, task-specific capability appraisal, and adaptive recovery under pressure. The model therefore moves beyond treating these constructs as interchangeable positive resources or parallel protective factors. Third, by connecting relational conditions, educational context, motivational judgments, and adaptive functioning, the model provides a cross-domain account of psychological wellbeing in vocational education. Its conceptual significance therefore lies not merely in arranging several variables in sequence, but in explaining how external support may acquire context-specific meaning and become associated with progressively more proximal forms of psychological functioning. Guided by this conceptual account, the present study examines whether perceived social support is associated with psychological wellbeing through the theoretically ordered roles of school belonging, academic self-efficacy, and psychological resilience among vocational college students.

## 2 Literature review and hypotheses

### 2.1 Theoretical grounding of the resource-transformation chain

The proposed serial mediation model is grounded in a resource-transformation perspective, with Conservation of Resources theory serving as the primary theoretical foundation. Conservation of Resources theory suggests that individuals strive to acquire, preserve, and build valued resources, and that the possession of one resource may facilitate the development of additional resources (Hobfoll, 1989; Hobfoll et al., 2018). This perspective provides the overarching resource-gain logic for explaining how perceived social support may be associated with psychological wellbeing through a sequence of school-contextual and personal psychological resources. Therefore, the term “resource-transformation chain” is not used in this study as a new standalone theory, but as an integrative process framework grounded in resource theory and informed by social support, school belonging, self-efficacy, and resilience perspectives.

Within this framework, Conservation of Resources theory provides the common logic linking the proposed sequence, but it does not by itself specify the psychological form that resource gain takes at each transition or prescribe a single fixed ordering among perceived social support, school belonging, academic self-efficacy, and psychological resilience. The complementary perspectives on belonging, self-efficacy, and resilience therefore do not function as separate explanations placed alongside COR theory; instead, they specify how the general resource-gain process may be expressed at successive stages of students' educational and psychological experience.

Perceived social support is conceptualized as an external relational resource because it reflects students' appraisal that emotional, informational, and practical assistance is available from important others (Zimet et al., 1988). From a belonging perspective, this relational availability may become embedded in the educational context when repeated experiences of acceptance, encouragement, and assistance shape how students interpret their position within the school community. School belonging therefore represents the school-contextual form through which students may experience themselves as accepted, included, respected, and valued within the educational environment (Goodenow, 1993; Slaten et al., 2016). Once relational support is experienced as connection with the school context, self-efficacy theory helps explain how this contextual resource may be associated with a more task-specific motivational resource. Students who feel accepted and connected may be more willing to participate in learning activities, seek feedback, collaborate with peers, and remain engaged in difficult academic and skills-related tasks. These experiences may provide mastery opportunities, social encouragement, and vicarious learning, which are important sources of academic self-efficacy (Bandura, 1997).

The transition from academic self-efficacy to psychological resilience reflects a further change in resource function. Academic self-efficacy concerns students' confidence in managing specific learning and skills-related demands, whereas psychological resilience concerns their broader capacity to maintain or restore functioning when stress and setbacks occur (Smith et al., 2008). Stronger efficacy beliefs may be associated with interpreting difficulties as manageable, sustaining effort, adjusting strategies, and re-engaging after failure. These appraisal and coping processes provide a psychological mechanism through which a task-specific motivational resource may become connected with a more proximal adaptive resource. Psychological resilience is consequently positioned closest to psychological wellbeing because it concerns the maintenance and restoration of psychological functioning under pressure. Accordingly, COR theory and the complementary perspectives form a cohesive explanation: COR theory provides the overarching logic of resource gain, while belonging, self-efficacy, and resilience perspectives specify how relational availability may become contextually embedded, linked with task-specific capability beliefs, and associated with adaptive recovery.

The vocational education context also gives the proposed resource-gain process a distinctive functional form. Because vocational learning is organized around teacher guidance, peer collaboration, workshop participation, and practicum communities, relational support may be closely connected with whether students experience themselves as accepted members of the educational environment. Because competence is repeatedly developed and evaluated through skills practice, practical assessment, and feedback, school belonging may be especially relevant to students' willingness to participate in efficacy-building experiences and to their confidence in managing occupationally relevant tasks. Moreover, repeated exposure to performance setbacks, practicum demands, and uncertainty surrounding the school-to-work transition may make efficacy-related appraisal, strategic adjustment, and re-engagement particularly relevant to psychological resilience. Resilience, in turn, is closely associated with psychological wellbeing because it concerns students' capacity to maintain or restore functioning under these recurring academic, practical, and transition-related pressures. Vocational education therefore serves as a theoretically meaningful context that shapes the functional relevance of each resource transition, rather than merely as the sampling setting of the study (Hong et al., 2021; Liu, 2026; Zhu et al., 2025).

### 2.2 Perceived social support and psychological wellbeing

Perceived social support refers to individuals' belief that supportive interpersonal resources are available when needed. It is conceptually different from received support because it emphasizes the subjective appraisal of support availability rather than the actual amount of help obtained from others (Haber et al., 2007). In the student context, perceived social support may come from family members, peers, teachers, classmates, and other significant relationships. Such support may provide emotional reassurance, a sense of being valued, and confidence that difficulties can be managed with the help of others. From a relational perspective, perceived support is psychologically meaningful because supportive relationships may help individuals regulate negative experiences and sustain positive functioning in daily life (Lakey and Orehek, 2011).

Psychological wellbeing reflects a positive state of psychological functioning rather than the mere absence of mental health problems. It is closely related to students' positive affect, life satisfaction, sense of meaning, and capacity to function effectively in their social and educational environments (Diener et al., 1999). For vocational college students, psychological wellbeing is especially relevant because their school experience is closely connected with applied learning, skills training, practicum requirements, and career preparation. These students may need to cope not only with general academic pressure, but also with practical training demands, occupational expectations, and uncertainty during the transition from school to work (Liu, 2026). Therefore, understanding the relational resources associated with their psychological wellbeing is important for explaining their positive adaptation in vocational education.

Existing research has provided theoretical and empirical support for the positive association between perceived social support and wellbeing (Hidalgo-Fuentes et al., 2024). Supportive relationships may facilitate thriving by helping individuals cope with adversity and pursue exploration, personal goals, and growth under non-adverse conditions (Feeney and Collins, 2015). Meta-analytic evidence also suggests that social support is positively related to wellbeing among young people, indicating that students who perceive stronger support tend to report more favorable psychological outcomes (Chu et al., 2010). In addition, wellbeing research has shown that positive psychological functioning is strongly embedded in social relationships, suggesting that students' subjective sense of being supported may be an important correlate of their overall wellbeing (Diener and Seligman, 2002).

In vocational college settings, perceived social support may be particularly important because students often face both school-based and career-oriented developmental tasks. In this context, perceived support may be associated with psychological wellbeing through two complementary processes: it may buffer the emotional impact of academic and career-related stress, while also reinforcing students' sense of being valued and capable of pursuing meaningful goals. By shaping how students appraise challenges, regulate negative emotions, and sustain engagement in learning and career preparation, the perceived availability of support may help maintain positive affect and effective psychological functioning. When students perceive that they are cared for and supported by important others, they may be more likely to interpret academic, practical, and career-related challenges as manageable. This perceived availability of support may help them maintain a more positive psychological state during learning and adaptation. Accordingly, the present study proposes that perceived social support is positively associated with psychological wellbeing among vocational college students. It should be noted that this hypothesis refers to the overall association between perceived social support and psychological wellbeing, rather than a direct effect after accounting for the proposed mediating mechanisms.

H1. Perceived social support is positively associated with psychological wellbeing among vocational college students.

### 2.3 Perceived social support and school belonging

School belonging refers to students' perceived connection with the school community, including feelings of being accepted, valued, included, and supported in the educational environment (Slaten et al., 2016). Recent higher education research has emphasized that belonging is a multidimensional construct involving students' connectedness to peers, staff, and the institution, their sense of safety and inclusion, and their perception of being valued and accepted (Allen et al., 2024). In the present study, school belonging is conceptualized as a school-contextual resource that links external relational support with students' subsequent academic and psychological resources. It is therefore different from psychological wellbeing itself: whereas psychological wellbeing reflects broader positive psychological functioning, school belonging captures students' perceived membership and connection within the school environment. Allen et al.'s (2024) review identifies belonging in higher education as shaped by quality relationships, inclusive environments, and institutional practices.

Perceived social support may be positively associated with school belonging because supportive relationships may signal to students that they are cared for, accepted, and not alone in dealing with school-related challenges (Li and Li, 2024). When students perceive support from family members, peers, teachers, or significant others, they may be more likely to interpret the school environment as safe, responsive, and personally meaningful. In this sense, perceived social support does not remain only an external relational resource; it may also be reflected in students' subjective connection with their educational environment. Recent evidence among undergraduate students showed that students with higher perceived social support reported stronger belonging to their institutional environment, and that sense of belonging mediated the relationship between perceived social support and academic persistence (Mtshweni, 2024).

This relationship may be especially important for vocational college students. Vocational education is characterized by applied learning, skills training, practicum requirements, teacher-student interaction, peer collaboration, and career-oriented preparation. Under such conditions, students' sense of belonging may depend strongly on whether they perceive support from proximal social relationships in both school and family contexts. In vocational education, this association may operate through relational affirmation and contextual interpretation. Support from teachers and peers may signal that students are legitimate and valued participants in classroom, workshop, and practicum communities, while support from family and significant others may reinforce the perception that their educational experiences are recognized and supported. Repeated experiences of acceptance and assistance may reduce feelings of exclusion and strengthen students' identification with the school community. Research on Chinese vocational school students found that parents' emotional support and school cooperation climate were positively associated with students' school belonging, which in turn was related to academic performance (Liu et al., 2022). Similarly, a study of secondary vocational students showed that social support was linked to school belonging in the mechanism explaining students' professional identity (Chen et al., 2023). More recent research on higher vocational college students also suggests that peer support is an important external resource for academic and social adjustment in vocational education contexts (Zhu et al., 2025).

Taken together, these findings suggest that perceived social support may provide a relational foundation for vocational college students' school belonging. When students perceive that important others are available to provide emotional reassurance, academic guidance, and practical help, they may feel more accepted and connected within their school community. Therefore, perceived social support is expected to be positively associated with school belonging among vocational college students. In the proposed model, school belonging is placed after perceived social support because it represents how an external relational resource may be manifested within the school context. That is, students who perceive stronger support from family members, peers, teachers, or significant others may be more likely to experience the school environment as accepting, responsive, and personally meaningful.

H2. Perceived social support is positively associated with school belonging among vocational college students.

### 2.4 School belonging and academic self-efficacy

School belonging may provide an important contextual foundation for students' academic self-efficacy (Korpershoek et al., 2020). Academic self-efficacy refers to students' beliefs about their capability to organize and complete academic tasks, persist in learning, and manage educational challenges. In the present study, it is conceptualized as an academic motivational belief that develops partly from students' experiences within the school environment. When students feel accepted, valued, and connected to their school community, they may be more willing to participate in learning activities, seek feedback, and interpret academic challenges as manageable rather than threatening. This supportive participation may contribute to academic self-efficacy through several mechanisms. Greater engagement may provide students with more opportunities to accumulate successful learning and skills-related experiences, while constructive feedback from teachers and peers may function as social encouragement regarding their capabilities. In addition, observing and collaborating with accepted peers may provide vicarious experiences that help students judge difficult academic and practical tasks as attainable.

Recent research supports the close relationship between belonging-related experiences and academic self-efficacy (van Lamoen et al., 2025; Ghadampour et al., 2025). For example, Yi et al. (2024) found that students' sense of belonging positively and significantly influenced academic self-efficacy in a sample of postgraduate students in Mainland China. Similarly, longitudinal evidence showed moderate to strong associations between school relatedness and academic self-efficacy (Wang et al., 2024a), suggesting that students' connection with the school environment is closely linked to their motivational functioning.

This relationship may be especially meaningful in vocational college contexts. Vocational college students are required to handle not only coursework, but also skills training, practical assessments, internship preparation, and career-oriented learning tasks (Yan, 2025). A stronger sense of school belonging may help these students perceive their learning environment as supportive and personally meaningful. Such belonging may increase their confidence in coping with academic and practice-related demands (Cai et al., 2023). Research on vocational students has also shown that academic self-efficacy is positively related to school and firm engagement, suggesting its importance for students' learning and internship-related adjustment (Hong et al., 2021).

This ordering is theoretically important because academic self-efficacy is treated as a task-specific motivational belief rather than as a general adaptive capacity. In vocational education, students' confidence in completing coursework, skills training, and assessment tasks is likely to develop from their participation in a supportive and meaningful school environment. Psychological resilience, by contrast, refers to students' broader capacity to recover from pressure and setbacks. Therefore, academic self-efficacy is positioned before psychological resilience in the present model: students may first develop confidence in handling learning-related tasks, and this confidence may then support their ability to adapt when they encounter difficulties.

Accordingly, school belonging can be understood as a school-contextual resource that may support stronger academic self-efficacy. When vocational college students feel that they are legitimate and valued members of the school community, they may be more likely to believe that they can successfully complete learning tasks and manage skills-related challenges. Therefore, the following hypothesis is proposed:

H3. School belonging is positively associated with academic self-efficacy among vocational college students.

### 2.5 Academic self-efficacy and psychological resilience

Academic self-efficacy refers to students' beliefs about their capability to manage learning tasks, meet academic requirements, and respond effectively to education-related challenges. From the perspective of self-efficacy theory, students with stronger efficacy beliefs are more likely to approach difficult tasks with confidence, sustain effort, and regard setbacks as manageable rather than as evidence of fixed personal inability (Bandura, 1997). Psychological resilience, in contrast, refers to a broader adaptive capacity to recover from stress, cope with setbacks, and maintain or restore functioning when difficulties occur (Gong et al., 2023). The proposed relationship between these constructs is therefore not based on treating them as interchangeable resources. Academic self-efficacy represents a task-specific capability belief, whereas psychological resilience concerns adaptive recovery across stressful situations.

Academic self-efficacy may be associated with psychological resilience first through students' appraisal of academic and skills-related difficulties. Students who believe that they are capable of managing a task may be more likely to interpret difficulty as a controllable challenge that can be addressed through effort, strategy, or assistance, rather than as an uncontrollable threat or proof of enduring incapacity. Such efficacy-related appraisals may preserve students' sense of agency when performance problems or setbacks occur and reduce the likelihood that temporary failure results in helplessness or withdrawal.

Second, these appraisals may be associated with coping and behavioral regulation. When students regard academic or practical demands as manageable, they may be more likely to persist, modify ineffective strategies, seek feedback or instrumental assistance when necessary, and re-engage after an unsuccessful attempt. Adaptive coping is closely related to students' capacity to remain functional under pressure and recover from difficulties (Wu et al., 2020). Academic self-efficacy may therefore be connected with resilience not simply because confident students experience fewer setbacks, but because they may respond to setbacks in ways that support continued functioning and recovery.

This mechanism may be particularly salient in vocational education, where students repeatedly encounter performance-based demands in coursework, skills training, practical assessment, practicum preparation, and career-related learning. Across these recurring situations, students may experience cycles of difficulty, strategic adjustment, renewed effort, and eventual recovery. Successful management of such task-specific setbacks may gradually reinforce a broader expectation that difficulties can be managed and that functioning can be restored after disruption. Repeated efficacy-consistent coping experiences may therefore provide a psychological basis through which confidence in managing concrete learning and training demands becomes associated with a more generalized capacity for adaptive recovery.

Existing evidence is consistent with this proposed relationship. Research involving Chinese vocational students has shown that self-efficacy and resilience are closely related and may operate together with other positive psychological resources in explaining adaptive functioning (Jiang, 2024; Zhang et al., 2024). Studies in broader educational contexts have similarly linked self-efficacy with psychological or academic resilience, suggesting that students' confidence in managing learning demands is associated with their capacity to adapt to pressure and recover from setbacks (Xu and Xu, 2025; Shengyao et al., 2024).

On this basis, academic self-efficacy is positioned before psychological resilience in the proposed sequence because efficacy-related appraisal, coping, and re-engagement processes may provide a psychological foundation for adaptive recovery. This ordering is conceptually specified rather than temporally established, and the cross-sectional design of the present study does not demonstrate that academic self-efficacy causally precedes psychological resilience. Accordingly, the following hypothesis is proposed:

H4. Academic self-efficacy is positively associated with psychological resilience among vocational college students.

### 2.6 Psychological resilience and psychological wellbeing

Psychological resilience refers to students' adaptive capacity to recover from stress, maintain functioning, and adjust positively when facing difficulties (Temiz and Comert, 2018). In the proposed resource-transformation chain, resilience is conceptualized as an adaptive psychological resource rather than as psychological wellbeing itself. This distinction is important because resilience explains how students may remain psychologically functional under pressure, whereas psychological wellbeing reflects broader positive psychological functioning and subjective adjustment.

Psychological resilience may be positively associated with psychological wellbeing because resilient students are more likely to regulate negative experiences, recover from setbacks, and maintain a sense of direction when encountering academic and life challenges (Xu et al., 2026a). In vocational college contexts, this adaptive capacity may be especially important. Vocational college students often face academic evaluation, skills training, practical assessments, internship preparation, and employment-related uncertainty. When students have stronger resilience, they may be better able to cope with these pressures and sustain more positive psychological functioning during the process of school adaptation and career preparation (Xu et al., 2026b).

Recent research provides support for the close relationship between resilience and wellbeing in student populations. A systematic review of resilience interventions among university students indicated that resilience-based programs are commonly associated with improvements in students' mental health and wellbeing (Abulfaraj et al., 2024). Evidence from university student samples also suggests that resilience is a salient personal resource associated with higher psychological wellbeing, even when other study-related and personal resources are considered (Bagdžiūnienė et al., 2025). In addition, recent work on academic resilience has shown that perseverance and adaptive coping in academic contexts are closely related to students' happiness and psychological wellbeing (Klutsey and Mahama, 2025). These findings suggest that resilience may help students maintain positive psychological functioning when facing academic, social, and developmental challenges.

In the resource-transformation chain, psychological resilience is positioned closest to psychological wellbeing because it concerns how students respond when stress and setbacks are actually encountered. School belonging and academic self-efficacy represent contextual and motivational resources that may prepare students to face challenges, whereas resilience reflects the more immediate adaptive processes through which students appraise setbacks as temporary and manageable, regulate negative emotions, restore engagement after disruption, and maintain goal-directed functioning. These processes may limit the accumulation of distress while preserving positive affect, vitality, and interest in daily life. Psychological resilience is therefore treated as the most proximal mediator because its functional content is directly related to maintaining or restoring the psychological functioning represented by wellbeing, while remaining conceptually distinct from psychological wellbeing itself.

Therefore, psychological resilience can be understood as a proximal adaptive resource associated with psychological wellbeing. For vocational college students, stronger resilience may help them respond more effectively to learning difficulties, training-related setbacks, and future career uncertainty. Accordingly, the following hypothesis is proposed:

H5. Psychological resilience is positively associated with psychological wellbeing among vocational college students.

### 2.7 The serial mediating roles of school belonging, academic self-efficacy, and psychological resilience

The preceding hypotheses suggest that perceived social support, school belonging, academic self-efficacy, psychological resilience, and psychological wellbeing are not isolated constructs, but may be connected through a theoretically ordered process. In this study, perceived social support is conceptualized as an external relational resource; school belonging represents students' school-contextual connection; academic self-efficacy reflects an academic motivational belief; and psychological resilience functions as an adaptive psychological resource. This order reflects a resource-transformation chain in which support perceived from important others may be linked to a stronger sense of connection with the school community, which may in turn be associated with greater confidence in managing academic and skills-related tasks and stronger adaptive capacity under pressure.

This serial process is particularly meaningful in vocational college contexts. Vocational college students' learning experiences are closely tied to applied learning, skills training, practical assessments, internship preparation, and future career transition. Under such conditions, perceived social support may not by itself fully explain the process through which students maintain psychological wellbeing. Instead, support may become psychologically meaningful when it helps students feel accepted and valued within their school environment. A stronger sense of school belonging may then provide a supportive basis for academic self-efficacy, because students who feel connected to the school community may be more willing to participate in learning, seek feedback, and believe that they can complete school- and training-related tasks. In turn, academic self-efficacy may be associated with stronger psychological resilience by helping students approach setbacks as manageable and recover more effectively from academic, practical, and career-related difficulties.

Within the Conservation of Resources framework, the proposed ordering is conceptually prioritized according to four related criteria: the source of each resource, its degree of contextual embeddedness, its functional specificity, and its proximity to students' responses to stress. Perceived social support is positioned first because it reflects the perceived availability of resources across important interpersonal relationships, including family members, friends, and significant others. It is therefore a relatively distal relational resource grounded in students' relationships with important others. School belonging follows because it represents the school-specific interpretation of whether students experience their educational environment as accepting, supportive, and personally meaningful. Academic self-efficacy is positioned after school belonging because it concerns students' task-specific judgments of their capability to manage academic and skills-related demands, which may be informed by participation, feedback, mastery opportunities, and vicarious learning within the school context. Psychological resilience follows academic self-efficacy because it concerns a broader and more proximal capacity to maintain or restore functioning when pressure and setbacks occur.

This functional progression also explains why the proposed ordering is conceptually preferable to several plausible alternatives. Placing school belonging before perceived social support would position a school-specific sense of membership as conceptually prior to the perceived availability of support across broader interpersonal relationships. Placing academic self-efficacy before school belonging would understate the contextual conditions that facilitate participation, feedback seeking, and other efficacy-building experiences. Placing psychological resilience before academic self-efficacy would position a generalized capacity for adaptive recovery as conceptually prior to students' appraisal that concrete learning and training demands are manageable. The proposed sequence is therefore not claimed to be the only possible pathway or an empirically established temporal process. Rather, it is theoretically prioritized as a progression from relational availability to contextual embedding, task-specific capability appraisal, adaptive recovery, and psychological wellbeing.

Recent research supports the plausibility of such sequential mechanisms. Evidence from vocational college students suggests that self-efficacy and resilience can operate as linked mediating resources between social support and student outcomes, indicating that external support may be associated with adaptive functioning through internal psychological resources (Zhang et al., 2024). Related evidence from higher education also shows that school belonging can mediate the association between perceived social support and students' academic persistence, suggesting that social support may first be reflected in students' connection with the educational environment, which may subsequently be associated with later adjustment processes (Mtshweni, 2024). Moreover, studies using serial mediation models in student populations have increasingly emphasized that wellbeing and adjustment are often shaped by multiple connected psychological mechanisms rather than by a single direct pathway (Wang et al., 2024b; Liang et al., 2025).

Integrating these perspectives, the present study proposes that school belonging, academic self-efficacy, and psychological resilience serially mediate the association between perceived social support and psychological wellbeing. This hypothesis highlights that the main contribution of the model is not merely to test whether perceived social support is associated with psychological wellbeing, but to examine how external relational support may be linked to school belonging, academic confidence, and resilience, which are in turn associated with psychological wellbeing.

H6. School belonging, academic self-efficacy, and psychological resilience serially mediate the association between perceived social support and psychological wellbeing among vocational college students.

## 3 Methodology

### 3.1 Participants and procedure

This study adopted a cross-sectional questionnaire design and used convenience sampling to recruit students currently enrolled in vocational colleges in Chongqing, Zhejiang Province, and Shanghai, China. Data were collected through an anonymous online questionnaire. With logistical assistance from teachers or student counselors at the participating institutions, the questionnaire link was distributed to eligible students, who completed the survey independently within a designated period. Before participation, students were informed of the purpose and procedures of the study. Participation was entirely voluntary, students could decline to participate or discontinue without penalty, and no compensation was provided.

A total of 500 questionnaires were collected. Responses were excluded if the questionnaire was incomplete, if the respondent failed the attention-check item, or if the responses showed evident straight-lining, such as selecting the same response option across all substantive questionnaire items. After data screening, 436 valid responses were retained for analysis, yielding a valid response rate of 87.2%.

The final sample consisted of 176 male students (40.4%), 254 female students (58.3%), and 6 students who selected “other/prefer not to answer” (1.4%). Participants ranged in age from 18 to 23 years, with a mean age of *M* = 19.41 years (*SD* = 1.36). In terms of year in college, 161 students were in their first year (36.9%), 148 were in their second year (33.9%), 117 were in their third year (26.8%), and 10 selected “other/prefer not to answer” (2.3%).

Participants came from a range of vocational fields. Specifically, 75 students majored in engineering and technical trades (17.2%), 85 in medicine and health sciences (19.5%), 74 in finance, business, and trade (17.0%), 75 in education (17.2%), 76 in culture and arts (17.4%), and 51 selected “other/prefer not to answer” (11.7%). Regarding urban/rural background, 250 students reported an urban background (57.3%) and 186 reported a rural background (42.7%). For self-rated family economic status, 61 students reported a relatively difficult status (14.0%), 245 reported an average status (56.2%), 99 reported a relatively good status (22.7%), and 31 reported a very good status (7.1%). The demographic characteristics of the participants are presented in Table 1.

**Table 1:** Demographic characteristics of participants.

Characteristic	Number (%)
Gender	
Male	176 (40.4)
Female	254 (58.3)
Other/prefer not to answer	6 (1.4)
Age	
18 years	126 (28.9)
19 years	118 (27.1)
20 years	107 (24.5)
21 years	53 (12.2)
22 years	19 (4.4)
23 years	13 (3.0)
Year in college	
First year	161 (36.9)
Second year	148 (33.9)
Third year	117 (26.8)
Other/prefer not to answer	10 (2.3)
Field of study	
Engineering and technical trades	75 (17.2)
Medicine and health sciences	85 (19.5)
Finance, business, and trade	74 (17.0)
Education	75 (17.2)
Culture and arts	76 (17.4)
Other/prefer not to answer	51 (11.7)
Urban/rural background	
Urban	250 (57.3)
Rural	186 (42.7)
Self-rated family economic status	
Relatively difficult	61 (14.0)
Average	245 (56.2)
Relatively good	99 (22.7)
Very good	31 (7.1)

*N* = 436. Percentages may not sum to 100 due to rounding. Age: *M* = 19.41, *SD* = 1.36, range = 18–23 years. Demographic variables were summarized descriptively and were not included as covariates in the structural model.

### 3.2 Measures

All measures were administered in Chinese. The questionnaire included five core constructs: perceived social support, school belonging, academic self-efficacy, psychological resilience, and psychological wellbeing. Unless otherwise specified, item scores were averaged to create composite scores for each construct. Negatively worded items were reverse-coded before computing scale scores. Higher scores indicated higher levels of the corresponding construct.

The questionnaire was prepared in Chinese for vocational college students. The measures were selected from established instruments and, where necessary, item wording was slightly adapted to fit the vocational college context, particularly for academic self-efficacy items referring to course learning, skills-related knowledge, and professional training demands. Where established Chinese-language items were available, these items were used directly. Any adaptations were limited to minor contextual wording intended to improve clarity for vocational college students and did not alter the substantive meanings of the original constructs. Negatively worded items in the school belonging and psychological resilience scales were reverse-coded before analysis. To improve transparency and replicability, the full Chinese questionnaire, including instructions, response anchors, demographic items, construct items, and the attention-check item, is provided in Supplementary material.

#### 3.2.1 Perceived social support

Perceived social support was measured using the 12-item Multidimensional Scale of Perceived Social Support (Zimet et al., 1988). The MSPSS assesses the extent to which individuals perceive support as available from three major interpersonal sources: family, friends, and significant others, and it has continued to be widely used in recent research on perceived social support and psychological wellbeing (Tannous-Haddad et al., 2024). This scale was suitable for the present study because vocational college students may rely on multiple relational resources when coping with academic pressure, skills-training demands, and career-related uncertainty.

Participants responded to each item on a 7-point Likert scale ranging from 1 to 7, with higher scores indicating stronger perceived social support. A sample item is “I can count on my friends when things go wrong.” The scale contains no reverse-coded items. In the present study, Cronbach's α for this scale was .939, indicating excellent internal consistency.

#### 3.2.2 School belonging

School belonging was assessed using a six-item school belonging scale adapted from the Programme for International Student Assessment framework (OECD, 2019; Palikara et al., 2025). The scale measures students' subjective sense of being accepted, included, and connected within the school environment. This construct is especially relevant to vocational college students because their learning experiences are embedded in school-based relationships, practical training contexts, peer interaction, and teacher–student communication.

Participants responded on a 4-point Likert scale, with higher scores representing stronger school belonging after reverse coding. A sample item is “I feel like I belong at school.” Negatively worded items, such as feeling like an outsider, feeling awkward and out of place, and feeling lonely at school, were reverse-coded before computing the mean score. In the present study, Cronbach's α for this scale was 0.817, indicating acceptable internal consistency.

#### 3.2.3 Academic self-efficacy

Academic self-efficacy was measured using eight items adapted from the self-efficacy component of the Motivated Strategies for Learning Questionnaire (Pintrich et al., 1991). The scale assesses students' confidence in understanding course content, completing academic tasks, and meeting learning requirements (Duncan and McKeachie, 2005). In the present study, the wording of the items was adapted to fit the vocational college context, where students are expected to manage both classroom learning and practice-oriented training.

Participants responded on a 7-point Likert scale ranging from 1 to 7, with higher scores indicating stronger academic self-efficacy. A sample item is “I am confident I can understand the course material.” No items were reverse-coded. In the present study, Cronbach's α for this scale was 0.915, indicating excellent internal consistency.

#### 3.2.4 Psychological resilience

Psychological resilience was assessed using the six-item Brief Resilience Scale (Smith et al., 2008). The BRS measures individuals' perceived ability to recover from stress and bounce back after difficulties, and recent validation studies among university and college students have continued to support its use as a brief measure of resilience (Minh Nguyen et al., 2024; Saha et al., 2025). This scale was appropriate for the present study because resilience was conceptualized as an adaptive psychological resource that may help vocational college students cope with academic setbacks, practical training difficulties, and school-to-work transition pressure.

Participants responded on a 5-point Likert scale ranging from 1 to 5, with higher scores indicating greater psychological resilience. A sample item is “I tend to bounce back quickly after hard times.” Following the standard scoring procedure, items 2, 4, and 6 were reverse-coded before calculating the mean score. In the present study, Cronbach's α for this scale was 0.834, indicating good internal consistency.

#### 3.2.5 Psychological wellbeing

Psychological wellbeing was measured using the five-item World Health Organization-Five wellbeing Index (WHO-5). The WHO-5 is a brief self-report measure designed to assess positive psychological wellbeing over the past 2 weeks (Topp et al., 2015; Fung et al., 2022). It captures positive mood, vitality, and general psychological functioning. This measure was suitable for the present study because it provides a concise assessment of students' recent positive psychological state rather than focusing on psychological distress.

Participants rated each item on a 0–5 scale, with higher scores indicating higher psychological wellbeing. A sample item is “I have felt cheerful and in good spirits.” The scale contains no reverse-coded items. Item scores were averaged for analysis. In the present study, Cronbach's α for this scale was 0.862, indicating good internal consistency.

### 3.3 Data analysis

Data analysis was conducted using SPSS 27.0.1 (IBM Corp., Armonk, NY, USA), AMOS 24 (IBM Corp., Armonk, NY, USA), and supplementary Python scripts (Python Software Foundation, Wilmington, DE, USA). Before the main analyses, the dataset was screened for completeness, invalid responses, and response patterns that could indicate low-quality data. After data screening, 436 valid questionnaires were retained for analysis. Negatively worded items were reverse-coded before computing scale scores. For each construct, item scores were averaged, with higher scores representing higher levels of the corresponding variable.

Demographic variables were summarized descriptively but were not included as covariates in the main structural model because the study did not propose hypotheses concerning demographic differences and aimed to maintain a focused test of the theoretically specified resource-transformation chain. The possible influence of demographic characteristics is acknowledged as a limitation and an issue for further investigation.

First, SPSS 27.0.1 was used to conduct descriptive statistics, reliability analysis, and Pearson correlation analysis. Means, standard deviations, observed score ranges, and Cronbach's α coefficients were calculated for perceived social support, school belonging, academic self-efficacy, psychological resilience, and psychological wellbeing. Pearson correlation coefficients were then computed to examine the bivariate associations among the five study variables. These analyses provided preliminary evidence for the direction and strength of associations before testing the measurement and structural models.

Second, confirmatory factor analysis (CFA) was conducted in AMOS 24 to evaluate the measurement model. The hypothesized five-factor model specified perceived social support, school belonging, academic self-efficacy, psychological resilience, and psychological wellbeing as five distinct latent constructs. To examine discriminant validity, the five-factor model was compared with several alternative measurement models, including four-factor, three-factor, two-factor, and single-factor models. Because all focal variables were measured using self-report questionnaires, common method bias was also assessed using a single-factor CFA approach. Specifically, the hypothesized five-factor measurement model was compared with a single-factor model in which all measurement items were loaded onto one general factor. Model fit was evaluated using multiple fit indices, including the chi-square/degrees of freedom ratio (χ^2^/*df*), comparative fit index (CFI), Tucker–Lewis index (TLI), root mean square error of approximation (RMSEA), and standardized root mean square residual (SRMR). In addition, standardized factor loadings were examined, and composite reliability (CR) and average variance extracted (AVE) were calculated to assess convergent validity.

Third, structural equation modeling (SEM) was performed in AMOS 24 to test the hypothesized serial mediation model. The structural model examined the theoretically ordered pathway from perceived social support to school belonging, academic self-efficacy, psychological resilience, and psychological wellbeing. Consistent with the hypotheses, H1 was treated as the overall association between perceived social support and psychological wellbeing, rather than as a direct-effect hypothesis after accounting for mediators. The main structural paths corresponding to H2–H5 were estimated, and the full serial mediation path corresponding to H6 was tested.

Finally, the mediating effects were examined using a bootstrap procedure. Bias-corrected 95% confidence intervals were calculated based on 5,000 bootstrap samples. An indirect effect was considered statistically significant when the 95% confidence interval did not include zero. The total indirect effect and the specific indirect effects were examined to determine whether school belonging, academic self-efficacy, and psychological resilience serially mediated the association between perceived social support and psychological wellbeing. Python scripts were used as an auxiliary reproducibility check to verify data scoring, descriptive statistics, reliability estimates, CR/AVE calculations, and indirect-effect estimates.

## 4 Research results

### 4.1 Descriptive statistics and correlations

Descriptive statistics, reliability coefficients, and Pearson correlations for the five study variables are presented in Table 2. The mean score of perceived social support was 5.170 (*SD* = 1.091), suggesting that participants generally reported a relatively high level of perceived support. The mean score of school belonging was 3.038 (*SD* = 0.598), indicating a moderate-to-high level of perceived connection with the school environment. Academic self-efficacy also showed a relatively high mean score (*M* = 5.106, *SD* = 1.122), suggesting that students generally held positive beliefs about their ability to manage academic and learning-related tasks. The mean score of psychological resilience was 3.589 (*SD* = 0.768), and the mean score of psychological wellbeing was 3.260 (*SD* = 1.013).

**Table 2:** Descriptive statistics and correlations among study variables.

Variable	Items	Min–Max	M	SD	1	2	3	4	5
1. PSS	12	1.08–7.00	5.170	1.091	1				
2. SB	6	1.17–4.00	3.038	0.598	0.508**	1			
3. ASE	8	1.50–7.00	5.106	1.122	0.412**	0.477**	1		
4. PR	6	1.17–5.00	3.589	0.768	0.346**	0.404**	0.542**	1	
5. PWB	5	0.00–5.00	3.260	1.013	0.365**	0.402**	0.469**	0.547**	1

*N* = 436. PSS, Perceived Social Support; SB, School Belonging; ASE, Academic Self-Efficacy; PR, Psychological Resilience; PWB, Psychological Wellbeing. Min–Max refers to the observed score range in the present sample. **p < 0.01 (two-tailed). All reported correlations were significant at *p* < 0.001.

The internal consistency of all five scales was acceptable to excellent. Cronbach's alpha coefficients ranged from 0.817 to .939. Specifically, perceived social support showed excellent reliability (α = 0.939), followed by academic self-efficacy (α = 0.915), psychological wellbeing (α = 0.862), psychological resilience (α = 0.834), and school belonging (α = 0.817).

Pearson correlation analysis showed that all five study variables were significantly and positively correlated with one another. Perceived social support was positively correlated with psychological wellbeing (*r* = 0.365, *p* < 0.001), providing preliminary support for H1. Perceived social support was also positively associated with school belonging (*r* = 0.508, *p* < 0.001), which was consistent with H2. In addition, school belonging was positively correlated with academic self-efficacy (*r* = 0.477, *p* < 0.001), academic self-efficacy was positively correlated with psychological resilience (*r* = 0.542, *p* < 0.001), and psychological resilience was positively correlated with psychological wellbeing (*r* = 0.547, *p* < 0.001). Overall, the correlation results were consistent with the hypothesized direction of the proposed serial mediation model.

### 4.2 Measurement model and construct validity

Before evaluating the hypothesized measurement model, common method bias was examined using a single-factor CFA approach because all focal variables were measured through self-report questionnaires. The single-factor model, in which all measurement items were loaded onto one general factor, showed poor fit to the data: χ^2^ = 3817.706, *df* = 629, χ^2^/*df* = 6.069, CFI = 0.609, TLI = 0.586, RMSEA = 0.108, and SRMR = 0.118. This fit was substantially worse than that of the hypothesized five-factor model. These results suggest that the observed relationships among the study variables were unlikely to be fully explained by a single common method factor. Nevertheless, the single-factor CFA represents only a limited diagnostic and cannot rule out all forms of common method variance; therefore, some residual risk of common method bias may remain. Confirmatory factor analysis was conducted to evaluate the measurement model. The hypothesized five-factor model specified perceived social support, school belonging, academic self-efficacy, psychological resilience, and psychological wellbeing as five distinct latent constructs. As shown in Table 3, the five-factor model demonstrated good model fit: χ^2^ = 690.825, *df* = 619, χ^2^/*df* = 1.116, *CFI* = 0.991, *TLI* = 0.991, *RMSEA* = 0.016, and *SRMR* = 0.032. These fit indices indicated that the proposed measurement model fit the data well.

**Table 3:** Fit indices for competing measurement models.

Model	χ^2^	df	χ^2^/*df*	CFI	TLI	RMSEA	SRMR
Five-factor model	690.825	619	1.116	0.991	0.991	0.016	0.032
Four-factor model	1008.969	623	1.620	0.953	0.949	0.038	0.042
Three-factor model	1502.158	626	2.400	0.893	0.886	0.057	0.071
Two-factor model	2940.177	628	4.682	0.717	0.700	0.092	0.111
Single-factor model	3817.706	629	6.069	0.609	0.586	0.108	0.118

CFI, comparative fit index; TLI, Tucker–Lewis index; RMSEA, root mean square error of approximation; SRMR, standardized root mean square residual. The five-factor model specified perceived social support, school belonging, academic self-efficacy, psychological resilience, and psychological wellbeing as separate latent constructs. In the four-factor model, psychological resilience and psychological wellbeing were combined. In the three-factor model, school belonging and academic self-efficacy were combined, and psychological resilience and psychological wellbeing were combined. In the two-factor model, perceived social support, school belonging, and academic self-efficacy were combined, and psychological resilience and psychological wellbeing were combined. In the single-factor model, all items loaded on one general factor. The single-factor model was also used as a CFA-based check for common method bias.

To further examine the distinctiveness of the five constructs, the five-factor model was compared with several alternative measurement models. The four-factor model, in which psychological resilience and psychological wellbeing were combined into one factor, showed acceptable but weaker fit than the five-factor model. The three-factor, two-factor, and single-factor models showed progressively poorer fit, especially in terms of CFI, TLI, RMSEA, and SRMR. These results provided evidence that the five-factor model offered the best representation of the data and that the five constructs were empirically distinguishable.

Convergent validity was further evaluated using standardized factor loadings, composite reliability (CR), and average variance extracted (AVE), following established measurement-model guidelines (Fornell and Larcker, 1981; Hair et al., 2019). As presented in Table 4, standardized factor loadings ranged from 0.694 to 0.788 for perceived social support, 0.590 to 0.701 for school belonging, 0.704 to 0.804 for academic self-efficacy, 0.607 to 0.722 for psychological resilience, and 0.710 to 0.775 for psychological wellbeing. All non-fixed factor loadings were statistically significant. The CR values ranged from 0.818 to 0.939, exceeding the commonly accepted threshold of 0.70 and indicating satisfactory composite reliability. The AVE values for perceived social support, academic self-efficacy, and psychological wellbeing exceeded 0.50.

**Table 4:** Standardized factor loadings, composite reliability, and average variance extracted.

Construct	Items	Standardized loading range	CR	AVE
Perceived social support	12	0.694–0.788	0.939	0.562
School belonging	6	0.590–0.701	0.818	0.429
Academic self-efficacy	8	0.704–0.804	0.915	0.575
Psychological resilience	6	0.607–0.722	0.834	0.457
Psychological wellbeing	5	0.710–0.775	0.862	0.555

CR, composite reliability; AVE, average variance extracted. Standardized loading range refers to the range of absolute standardized factor loadings for each construct in the five-factor measurement model.

Although the AVE values for school belonging (AVE = 0.429) and psychological resilience (AVE = 0.457) were below the recommended threshold of 0.50, this finding indicates that these latent constructs accounted for less than half of the average variance in their indicators and that their convergent validity should therefore be interpreted with caution. Nevertheless, both constructs were retained because their CR values exceeded 0.80, all non-fixed factor loadings were statistically significant, and the hypothesized five-factor model demonstrated good fit and outperformed the alternative measurement models. Taken together, the results indicated satisfactory composite reliability and generally acceptable convergent validity, although the convergent validity of school belonging and psychological resilience warrants cautious interpretation. Overall, the CFA results supported the adequacy of the measurement model for subsequent structural analyses.

### 4.3 Structural model and hypothesis testing

The hypothesized structural model was tested using structural equation modeling. The model included the serial pathway from perceived social support to school belonging, academic self-efficacy, psychological resilience, and psychological wellbeing, together with the direct path from perceived social support to psychological wellbeing. The structural model showed good fit to the data: χ^2^ = 726.643, *df* = 624, χ^2^/*df* = 1.164, CFI = 0.987, TLI = 0.987, RMSEA = 0.019, and SRMR = 0.053. These indices indicated that the hypothesized structural model provided an acceptable representation of the observed data.

**Figure 1:**
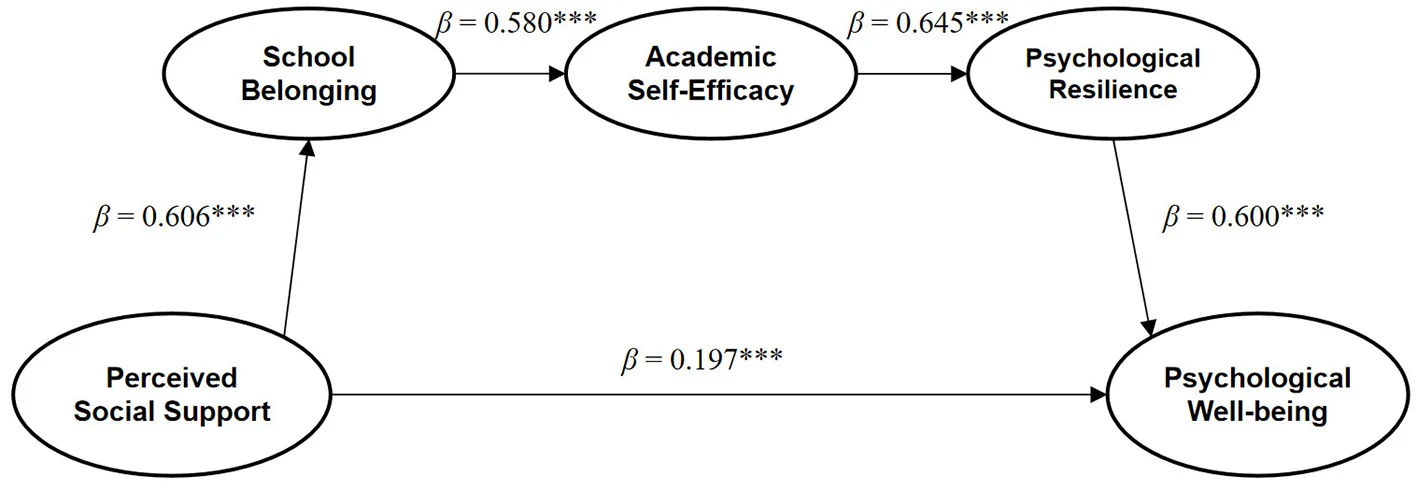
Structural model results. Standardized coefficients are shown. ***p < 0.001. The figure displays the hypothesized serial paths and the direct path from perceived social support to psychological wellbeing.

The standardized estimates for the hypothesized serial paths and the direct path from perceived social support to psychological wellbeing are displayed in Figure 1. Perceived social support was positively associated with school belonging (β = 0.606, *p* < 0.001), supporting H2. School belonging was positively associated with academic self-efficacy (β = 0.580, *p* < 0.001), supporting H3. Academic self-efficacy was positively associated with psychological resilience (β = 0.645, *p* < 0.001), supporting H4. Psychological resilience was positively associated with psychological wellbeing (β = 0.600, *p* < 0.001), supporting H5.

The direct path from perceived social support to psychological wellbeing remained statistically significant after school belonging, academic self-efficacy, and psychological resilience were included in the model (β = 0.197, *p* < 0.001). This result should be interpreted in relation to the hypothesis structure of the present study. H1 refers to the overall association between perceived social support and psychological wellbeing rather than only the direct effect after accounting for mediators. Therefore, the significant direct path provides additional evidence that perceived social support was positively associated with psychological wellbeing, while the mediation analysis further examines whether the proposed serial indirect pathway was also statistically significant.

Overall, the structural model results were consistent with the hypothesized path sequence from perceived social support to school belonging, academic self-efficacy, psychological resilience, and psychological wellbeing. The significant direct path from perceived social support to psychological wellbeing also suggests the possibility of partial mediation, which is further examined through the bootstrap mediation analysis reported in the next section.

### 4.4 Mediation analysis

The mediating effect of school belonging, academic self-efficacy, and psychological resilience was examined using a bootstrap procedure with 5,000 resamples. Bias-corrected 95% confidence intervals were used to determine the significance of indirect effects. An effect was considered statistically significant when the confidence interval did not include zero.

As shown in Table 5, the total effect of perceived social support on psychological wellbeing was significant [β = 0.333, 95% BC*CI* [0.234, 0.419]]. The direct effect of perceived social support on psychological wellbeing also remained significant after school belonging, academic self-efficacy, and psychological resilience were included in the model [β = 0.197, 95% BC*CI* [0.104, 0.288]]. These results indicate that perceived social support was positively associated with psychological wellbeing both overall and directly.

**Table 5:** Bootstrap results for mediation effects.

Effect	Standardized estimate	95% BC CI	Conclusion
Direct effect: PSS → PWB	0.197	[0.104, 0.288]	Significant
Serial indirect effect: PSS → SB → ASE → PR → PWB	0.136	[0.096, 0.185]	Significant
Total indirect effect	0.136	[0.096, 0.185]	Significant
Total effect	0.333	[0.234, 0.419]	Significant

*N* = 436. PSS, Perceived Social Support; SB, School Belonging; ASE, Academic Self-Efficacy; PR, Psychological Resilience; PWB, Psychological Wellbeing. BC CI, bias-corrected confidence interval. Bootstrap estimates were based on 5,000 resamples. The mediation results in the main hypothesized model are reported for the theoretically specified full serial pathway. Additional plausible indirect pathways were examined in a supplementary expanded model as a robustness check.

More importantly, the serial indirect effect through school belonging, academic self-efficacy, and psychological resilience was significant [β = 0.136, 95% BC*CI* [0.096, 0.185]]. Because the confidence interval did not include zero, H6 was supported. Specifically, perceived social support was associated with higher school belonging, which was in turn associated with stronger academic self-efficacy, greater psychological resilience, and ultimately higher psychological wellbeing. Since both the direct effect and the serial indirect effect were significant, the results indicated a partial serial mediation pattern rather than a complete mediation pattern.

Overall, the mediation results were consistent with the proposed resource-transformation chain, suggesting that vocational college students' perceived social support was associated with psychological wellbeing partly through school belonging, academic self-efficacy, and psychological resilience. To address the possibility that other theoretically plausible indirect pathways may also exist, an expanded mediation model was further estimated as a robustness check. In this supplementary model, additional paths were allowed from perceived social support to academic self-efficacy and psychological resilience, from school belonging to psychological resilience and psychological wellbeing, and from academic self-efficacy to psychological wellbeing. This model was not used to replace the hypothesized serial mediation model, but to examine whether the full serial indirect pathway remained meaningful after accounting for alternative shorter pathways. The detailed results are reported in Supplementary Tables S1, S2.

## 5 Discussion

### 5.1 Major findings

This study examined how perceived social support was associated with psychological wellbeing among vocational college students through school belonging, academic self-efficacy, and psychological resilience. The findings suggest that perceived support may have both immediate and resource-linked significance for students' psychological functioning. Feeling that care, encouragement, and assistance are available from important others may provide emotional reassurance, reinforce students' sense of being valued, and help them appraise academic and career-related demands as more manageable. The partial mediation pattern further suggests that the association between perceived social support and psychological wellbeing cannot be reduced entirely to the three proposed mediators. Perceived support may also be associated with wellbeing through other relational or emotional processes not directly represented in the present model, which is consistent with research emphasizing both the stress-buffering and thriving-promoting functions of supportive relationships (Chu et al., 2010; Feeney and Collins, 2015).

More importantly, the serial indirect pattern provides a process-oriented explanation of how external relational support may be connected with students' positive psychological functioning. Perceived support may first become embedded in students' educational experience when supportive interactions with family members, teachers, peers, and other significant persons are associated with students interpreting the school environment as accepting, responsive, and personally meaningful. A stronger sense of school belonging may then encourage participation, feedback seeking, and engagement in academic and skills-related activities, thereby providing opportunities for successful experiences, social encouragement, and peer learning that are relevant to academic self-efficacy (Bandura, 1997). Confidence in managing concrete learning and training demands may, in turn, be associated with greater persistence, strategic adjustment, and re-engagement after failure. Psychological resilience represents the most proximal resource in this sequence because it concerns students' capacity to regulate negative experiences and restore functioning when setbacks are encountered. The mediators therefore appear to occupy different functional positions rather than operating as interchangeable protective factors.

This interpretation is particularly relevant to vocational education, where students must manage classroom learning together with skills training, practical assessment, practicum preparation, and uncertainty about the transition from school to work. Under these conditions, social support may be especially meaningful when it is reflected in students' connection with the school community, confidence in handling occupationally relevant learning tasks, and capacity to recover from training- and career-related setbacks. The supplementary expanded mediation model also showed that the hypothesized full serial indirect pathway remained significant when several plausible shorter pathways were included (see Supplementary Tables S1, S2). This finding suggests that the proposed sequence should not be regarded as the only possible mechanism, but as a theoretically meaningful pathway that remained evident alongside other associations. Given the cross-sectional design, however, the proposed ordering should be interpreted as theoretically specified rather than as evidence of established temporal causality.

### 5.2 Theoretical contributions

This study makes three theoretical contributions that increase the explanatory specificity of the proposed resource-transformation framework rather than merely restating the structure of the serial mediation model. First, the study refines the application of Conservation of Resources theory to students' psychological wellbeing. COR theory proposes that access to valued resources may facilitate the acquisition or strengthening of additional resources (Hobfoll, 1989; Hobfoll et al., 2018), but this general proposition does not specify the psychological form that resource gains may take across different domains of students' experience. The present framework adds process-level specificity by distinguishing relational availability, contextual embeddedness, task-specific capability appraisal, and adaptive recovery as functionally different expressions of resource gain. The contribution is therefore not the proposal of a new resource theory, but a more differentiated account of how COR's general resource-gain logic may be expressed across interpersonal, educational, motivational, and adaptive domains.

Second, the study contributes a functionally differentiated understanding of positive psychological resources. The proposed mediators are not treated as interchangeable protective factors whose effects simply accumulate. School belonging represents the contextual embedding through which perceived relational support may acquire meaning within the educational environment. Academic self-efficacy represents students' task-specific judgments that academic and skills-related demands are manageable, whereas psychological resilience represents the more proximal capacity to maintain or restore functioning when setbacks occur. The complementary perspectives on belonging, self-efficacy, and resilience therefore specify distinct psychological expressions of the overarching COR process. Conceptually, this framework suggests that the adaptive significance of resource gain depends not only on the availability of multiple resources, but also on how resources with different functions become connected across successive stages of students' adaptation.

Third, the study positions vocational education as a theoretically meaningful context that shapes the functional relevance of the proposed resource process. Vocational learning is relationally embedded in teacher guidance, peer collaboration, workshop participation, and practicum communities; it is also performance- and feedback-intensive because competence is repeatedly developed and evaluated through skills practice and practical assessment. Moreover, students face recurring setbacks and uncertainty during practicum preparation and the transition from school to work. These characteristics help explain why relational support may be especially relevant when it is embedded in school belonging, why belonging may facilitate efficacy-building experiences, and why task-specific capability beliefs may be closely associated with adaptive recovery. The vocational education context therefore informs how resource gains may be psychologically expressed rather than merely providing a different population in which to test the model.

Taken together, the proposed sequence advances existing theoretical understanding in three related respects. It moves beyond viewing resources as independent or parallel protective factors, distinguishes positive psychological resources according to the different functions they perform in students' adaptation, and incorporates educational context into the explanation of how resource gains may acquire psychological meaning. Psychological wellbeing is therefore conceptualized not simply as an outcome associated with the accumulation of beneficial resources, but as related to a contextually embedded and functionally differentiated process connecting relational availability, contextual membership, task-specific capability appraisal, and adaptive recovery. This interpretation represents a theoretical specification of the proposed process rather than evidence that the cross-sectional findings establish a single fixed temporal or causal sequence.

### 5.3 Practical implications

The findings suggest that vocational colleges may benefit from adopting a multilevel approach to student wellbeing rather than relying only on general psychological counseling. Because perceived social support, school belonging, academic self-efficacy, and psychological resilience occupy different functional positions, practical initiatives could address both students' external relational environments and their internal academic and adaptive resources. Such initiatives may be particularly valuable during periods involving entry into college, practical assessment, internship preparation, and the transition from school to work.

First, colleges could strengthen students' perceived social support and school belonging through structured and sustained relational practices. Teacher mentoring, regular counselor check-ins, peer-mentoring groups, and accessible channels for academic and emotional assistance may help students perceive that support is available when needed. Family-school communication may also be useful when students experience difficulties related to study, practical training, or career decisions. To strengthen school belonging, institutions could organize stable learning or training groups, inclusive orientation activities, collaborative workshop tasks, and opportunities for students to participate in professional or campus communities. Teachers should also attend to signs of social exclusion and ensure that students receive respectful communication, recognition, and opportunities to contribute. These strategies are consistent with research emphasizing the roles of supportive relationships, inclusive institutional practices, and peer support in students' sense of belonging and adjustment (Allen et al., 2024; Zhu et al., 2025).

Second, academic self-efficacy could be supported by designing learning and skills-training experiences that provide attainable mastery opportunities. Complex vocational tasks could be divided into progressive stages, with clear performance criteria and timely formative feedback. Teachers could demonstrate effective strategies, use competent peers as models, and help students identify specific improvements after unsuccessful attempts rather than interpreting failure as evidence of fixed inability. Academic coaching and career-preparation guidance could also connect classroom and practical achievements with students' confidence in completing future occupational tasks. These practices correspond to the importance of mastery experiences, social encouragement, and vicarious learning in the development of self-efficacy (Bandura, 1997).

Third, resilience support could be integrated into ordinary academic, practical-training, and career-preparation activities rather than being offered only after serious difficulties arise. Colleges could provide workshops on stress appraisal, emotion regulation, coping planning, and recovery from setbacks, together with guided reflection following practical-assessment failure, internship difficulties, or employment-related disappointment. Counselors and teachers could help students distinguish temporary setbacks from stable personal inadequacy, identify available resources, and develop concrete re-engagement plans. Students experiencing persistent or severe distress should also be referred to professional psychological services. Systematic-review evidence suggests that resilience-oriented interventions may be associated with improvements in university students' mental health and wellbeing (Abulfaraj et al., 2024). However, because the present study was cross-sectional, these recommendations should be understood as theory-informed practical implications rather than as intervention effects directly established by the current findings.

### 5.4 Limitations and future directions

Several limitations should be acknowledged. First, this study used a cross-sectional questionnaire design. Although the hypothesized serial mediation model was theoretically grounded and statistically supported, the cross-sectional nature of the data does not allow causal conclusions to be drawn. The observed associations among perceived social support, school belonging, academic self-efficacy, psychological resilience, and psychological wellbeing should therefore be interpreted as statistical relationships rather than evidence of temporal or causal effects. Future studies could use longitudinal designs to examine whether perceived social support predicts later changes in school belonging, academic self-efficacy, resilience, and psychological wellbeing over time.

Second, the study relied on self-report measures. Although the single-factor CFA model fitted the data substantially worse than the hypothesized five-factor model, this diagnostic cannot rule out all forms of common method variance. Self-report data may also be influenced by social desirability and individual response tendencies. Future research could combine self-report questionnaires with teacher ratings, peer evaluations, behavioral indicators, or school administrative data to provide a more comprehensive assessment of students' adaptation and wellbeing.

Third, although the sample included vocational college students from Zhejiang, Chongqing, and Shanghai, the findings may not be fully generalizable to all vocational college students in China. Vocational education settings may differ across regions, institutions, majors, and training systems. Future studies should include broader and more diverse samples, especially students from different provinces, school types, and vocational disciplines, to test whether the proposed resource-transformation chain is stable across contexts.

Fourth, although demographic characteristics such as gender, year in college, field of study, urban/rural background, and family economic status were collected and reported descriptively, they were not included as covariates in the main structural model. This decision was made because the study did not propose hypotheses concerning demographic differences and aimed to maintain a focused test of the theoretically specified resource-transformation chain. Nevertheless, future research could examine whether the proposed serial mediation process remains stable after accounting for demographic differences, or whether it varies across different student groups.

Finally, although the supplementary expanded mediation model examined several plausible shorter pathways and showed that the hypothesized full serial indirect pathway remained significant, the analysis did not cover all possible mechanisms or alternative orderings among the constructs. The proposed sequence should therefore be understood as a theoretically meaningful pathway rather than as the only possible process linking perceived social support with psychological wellbeing. Other factors, such as teacher support, peer support, career adaptability, academic stress, internship experience, and employment anxiety, may also play important roles in vocational college students' psychological wellbeing. Future studies could compare alternative theoretically plausible models and extend the present framework by incorporating additional school, family, and career-related factors.

## 6 Conclusion

This study examined the relationship between perceived social support and psychological wellbeing among vocational college students, with a focus on the serial mediating roles of school belonging, academic self-efficacy, and psychological resilience. The findings were consistent with the proposed resource-transformation chain. Perceived social support was positively associated with psychological wellbeing, and the sequential pathway from perceived social support to school belonging, academic self-efficacy, psychological resilience, and psychological wellbeing was statistically significant.

The results suggest that vocational college students' psychological wellbeing is not only related to the amount of support they perceive from important interpersonal sources, but also to how such support is connected with school-contextual and personal psychological resources. Specifically, perceived social support was associated with students' sense of connection to the school environment; stronger school belonging may be associated with greater academic self-efficacy; academic self-efficacy may further relate to higher psychological resilience; and resilience may be associated with better psychological wellbeing. Since both the direct effect and the serial indirect effect were significant, the findings indicate a partial serial mediation pattern.

Overall, this study contributes to the literature by offering a context-sensitive explanation of psychological wellbeing among vocational college students. It highlights the importance of integrating relational support, school belonging, academic confidence, and resilience when understanding students' positive psychological functioning. These findings also suggest that vocational colleges should not only strengthen students' external support systems, but also foster school belonging, academic self-efficacy, and psychological resilience to better support students' wellbeing during applied learning, skills training, and school-to-work transition.

## Data Availability

The raw data supporting the conclusions of this article will be made available by the authors, without undue reservation.
