# Supplementary material for: Perceived social support and psychological wellbeing among vocational college students: the serial mediating roles of school belonging, academic self-efficacy, and psychological resilience
Source: Front Psychol. 2026 Aug 27;17:1894510. doi: 10.3389/fpsyg.2026.1894510 (PMC13557995; doi:10.3389/fpsyg.2026.1894510)
Supplement: Supplementary file 1 [file Data_Sheet_1.pdf]

Supplementary Analysis: Expanded Mediation Model

To examine whether the hypothesized full serial mediation pathway remained meaningful when other theoretically plausible pathways were considered, we estimated an expanded mediation model as a supplementary robustness check. In this model, the hypothesized paths from perceived social support to school belonging, from school belonging to academic self-efficacy, from academic self-efficacy to psychological resilience, and from psychological resilience to psychological well-being were retained. In addition, several theoretically plausible shorter paths were allowed, including paths from perceived social support to academic self-efficacy and psychological resilience, from school belonging to psychological resilience and psychological well-being, and from academic self-efficacy to psychological well-being.

The results showed that the hypothesized core paths remained significant. Among the additional shorter paths, perceived social support was significantly associated with academic self-efficacy, and school belonging was significantly associated with psychological resilience. However, the direct path from perceived social support to psychological well-being was not significant in the expanded model. The bootstrap analysis further showed that the hypothesized full serial indirect pathway from perceived social support to psychological well-being through school belonging, academic self-efficacy, and psychological resilience remained significant. These results suggest that the proposed resource-transformation chain should not be interpreted as the only possible mechanism, but as a theoretically meaningful pathway that remained robust when alternative shorter pathways were also considered.

Supplementary Table S1. Structural paths in the expanded mediation model

Path	β	SE	z	p	Conclusion
PSS → SB	0.585	0.031	9.528	< .001	Significant
PSS → ASE	0.192	0.060	3.213	.001	Significant
SB → ASE	0.434	0.138	6.312	< .001	Significant
PSS → PR	0.063	0.039	1.064	.287	Not significant
SB → PR	0.182	0.093	2.597	.009	Significant
ASE → PR	0.495	0.042	7.729	< .001	Significant
PSS → PWB	0.091	0.047	1.609	.108	Not significant
SB → PWB	0.125	0.112	1.837	.066	Not significant
ASE → PWB	0.123	0.053	1.908	.056	Not significant
PR → PWB	0.478	0.088	6.724	< .001	Significant

Note: Paths with p values greater than .05 were not interpreted as statistically significant.

Supplementary Table S2. Bootstrapped indirect effects in the supplementary expanded mediation model

Indirect pathway	β	95% BC CI	Conclusion
PSS → SB → PWB	0.073	[-.009, .159]	Not significant
PSS → ASE → PWB	0.024	[.0003, .064]	Significant but small in magnitude
PSS → PR → PWB	0.030	[-.025, .089]	Not significant
PSS → SB → ASE → PWB	0.031	[-.002, .071]	Not significant
PSS → SB → PR → PWB	0.051	[.013, .099]	Significant
PSS → ASE → PR → PWB	0.045	[.017, .087]	Significant
PSS → SB → ASE → PR → PWB	0.060	[.036, .095]	Significant
Total indirect effect	0.314	[.222, .409]	Significant
Direct effect: PSS → PWB	0.091	[-.022, .205]	Not significant
Total effect	0.404	[.309, .491]	Significant

Supplementary Material 1. Chinese questionnaire used in the present study

职业院校学生感知社会支持与心理幸福感调查问卷

正式发放版

知情同意与填写说明

您好！感谢您参与本次问卷调查。本问卷仅用于学术研究，采用匿名方式填写，所有数据只用于统计分析，不会用于评价个人表现。

本问卷答案没有对错之分，请根据您的真实感受独立作答。预计填写时间约为 8-10 分钟。

如果您愿意参与，请继续填写；如果不愿意，可以随时停止作答。

**我已阅读以上说明，并自愿参与本研究。**	□ **同意**

一、基本信息

**1. 性别**	□ 男 □ 女 □ 其他/不愿说明
**2. 年龄**	____ 岁
**3. 年级**	□ 一年级 □ 二年级 □ 三年级 □ 其他：_______
**4. 专业类别**	□ 理工类 □ 医药卫生类 □ 财经商贸类 □ 教育类 □ 文化艺术类 □ 其他：_______
**5. 城乡背景**	□ 城镇 □ 农村
**6. 家庭经济状况自评**	□ 较困难 □ 一般 □ 较好 □ 很好

二、正式题项

请认真阅读每个陈述，并根据您的实际情况填写对应数字。每题只能选择一个答案。

不同模块的选项范围可能不同，请先阅读每个模块前的作答说明。

A. 感知社会支持

请根据您的真实感受填写数字：1=非常不同意，2=很不同意，3=稍不同意，4=中立，5=稍同意，6=很同意，7=非常同意。

题项	作答（填数字）
A1. 当我需要帮助时，我身边有重要的人可以依靠。	____
A2. 我有重要的人可以分享快乐和困难。	____
A3. 当我需要时，我的家人会给我实际帮助。	____
A4. 我能从家人那里获得情感上的帮助与支持。	____
A5. 我有重要的人能够安慰和鼓励我。	____
A6. 当我遇到困难时，朋友会尽力帮助我。	____
A7. 当事情不顺利时，我可以依靠朋友。	____
A8. 我可以和家人谈论自己的问题。	____
A9. 我有朋友可以分享自己的感受。	____
A10. 有重要的人关心我的感受。	____
A11. 我的家人愿意帮助我做决定。	____
A12. 我可以和朋友讨论自己遇到的困难。	____

B. 学校归属感

请根据您的真实感受填写数字：1=非常不同意，2=不同意，3=同意，4=非常同意。

题项	作答（填数字）
B1. 我有时觉得自己在学校被排除在外。	____
B2. 我在学校比较容易交到朋友。	____
B3. 我觉得自己属于这所学校。	____
B4. 我有时觉得自己在学校不自在或格格不入。	____
B5. 学校里的其他同学似乎接纳并喜欢我。	____
B6. 我有时在学校感到孤独。	____

C. 学业自我效能

请根据您的真实感受填写数字：1=完全不符合，2=很不符合，3=较不符合，4=一般，5=较符合，6=很符合，7=非常符合。

题项	作答（填数字）
C1. 我相信自己能够掌握课程中教授的知识和技能。	____
C2. 我有信心理解课程中较难的内容。	____
C3. 我相信自己能够较好地完成课程作业和考核。	____
C4. 我预期自己能在当前课程中取得较好成绩。	____
C5. 我相信自己能够理解课程中的基本概念。	____

题项	作答（填数字）
C6. 我有信心理解教师讲授的复杂知识点。	___
C7. 与同学相比，我相信自己能在学习任务中表现良好。	___
C8. 我确信自己能够学好本专业所要求的知识和技能。	___

D. 心理韧性

请根据您的真实感受填写数字：1=非常不同意，2=不同意，3=一般，4=同意，5=非常同意。

题项	作答（填数字）
D1. 经历困难后，我通常能较快恢复过来。	___
D2. 对我来说，度过有压力的事件比较困难。	___
D3. 在经历压力后，我通常不需要太长时间就能恢复。	___
D4. 当不好的事情发生后，我很难恢复到正常状态。	___
D5. 我通常能够较顺利地度过困难时期。	___
D6. 我往往需要很长时间才能从挫折中恢复过来。	___

E. 心理幸福感

请根据过去两周的实际感受填写数字：0=从不，1=偶尔，2=少于一半时间，3=多于一半时间，4=大多数时间，5=一直。

题项	作答（填数字）

题项	作答（填数字）
E1. 在过去两周，我感到心情愉快、精神良好。	____
E2. 在过去两周，我感到平静和放松。	____
E3. 在过去两周，我感到积极、有活力。	____
E4. 在过去两周，我醒来时感到精神恢复良好。	____
E5. 在过去两周，我的日常生活中有让我感兴趣的事情。	____

F. 作答认真度检测

请根据题目要求作答。

题项	作答（填数字）
F1. 为保证作答质量，请在本题选择“4=同意”。	____

问卷到此结束。再次感谢您的支持与配合！
